# Circulating MicroRNAs in Elderly Type 2 Diabetic Patients

**DOI:** 10.1155/2018/6872635

**Published:** 2018-04-10

**Authors:** Giuseppina Catanzaro, Zein Mersini Besharat, Martina Chiacchiarini, Luana Abballe, Claudia Sabato, Alessandra Vacca, Paola Borgiani, Francesco Dotta, Manfredi Tesauro, Agnese Po, Elisabetta Ferretti

**Affiliations:** ^1^Department of Experimental Medicine, Sapienza University of Rome, Rome, Italy; ^2^Department of Molecular Medicine, Sapienza University of Rome, Rome, Italy; ^3^Department of Biomedicine and Prevention, Tor Vergata University, Rome, Italy; ^4^Fondazione Umberto Di Mario ONLUS, Rome, Italy; ^5^Hypertension and Nephrology Unit, Department of Systems Medicine, Tor Vergata University, Rome, Italy

## Abstract

The circulating microRNAs (miRNAs) associated with type 2 diabetes (T2D) in elderly patients are still being defined. To identify novel miRNA biomarker candidates for monitoring responses to sitagliptin in such patients, we prospectively studied 40 T2D patients (age > 65) with HbA1c levels of 7.5–9.0% on metformin. After collection of baseline blood samples (*t*_0_), the dipeptidyl peptidase-IV (DPP-IV) inhibitor (DPP-IVi) sitagliptin was added to the metformin regimen, and patients were followed for 15 months. Patients with HbA1c < 7.5% or HbA1c reduction > 0.5% after 3 and 15 months of therapy were classified as “responders” (group R, *n* = 34); all others were classified as “nonresponders” (group NR, *n* = 6). Circulating miRNA profiling was performed on plasma collected in each group before and after 15 months of therapy (*t*_0_ and *t*_15_). Intra- and intergroup comparison of miRNA profiles pinpointed three miRNAs that correlated with responses to sitagliptin: miR-378, which is a candidate biomarker of resistance to this DPP-IVi, and miR-126-3p and miR-223, which are associated with positive responses to the drug. The translational implications are as immediate as evident, with the possibility to develop noninvasive diagnostic tools to predict drug response and development of chronic complications.

## 1. Introduction

With >400 million patients worldwide, type 2 diabetes (T2D) is among the most frequently diagnosed metabolic disorders. T2D is a multifactorial disease: genetic, lifestyle, and environmental factors combine to render target tissues insensitive to insulin, resulting in increasingly high blood levels of glucose. The disease is associated with serious and frequently disabling long-term complications, including cardiovascular disease, renal failure, neuropathy, and blindness, and it is therefore one of the leading causes of the global increase in morbidity and mortality [1–3]. Outcomes could be improved by earlier diagnosis, while the disease is still in the initial phase, and more rational use of currently available therapies (i.e., targeting drugs to the patients most likely to benefit from them). For this reason, there is an urgent need for new biomarkers with potential applications in the prevention and early diagnosis of T2D and for predicting its response to therapy, especially for fragile elderly patients [1, 2, 4].

Recently, microRNAs (miRNAs)—short (21-22 nucleotides), single-stranded, noncoding RNAs—have been detected in human plasma and other biological fluids, and in some cases, their expression profiles prove to be disease-specific [5–9]. Compared with many more conventional biomarkers, miRNAs offer several advantages, such as high stability, even under drastic conditions. In addition, miRNAs have been identified as major regulators of pancreatic *β*-cell mass and function, that is, the two key factors in the pathogenesis of T2D [9–11]. For these reasons, they are considered a promising source of biomarkers for diagnosing and staging T2D as well as for predicting their response to therapy [1, 12].

Glucagon-like peptide-1 (GLP-1) is an incretin hormone that stimulates insulin release by *β*-cells in a glucose-dependent manner and, at the same time, reduces glucagon secretion by alpha-cells. GLP-1-based therapeutic strategies have an increasing role in T2D treatment and consist of GLP-1 agonists and dipeptidyl peptidase-IV (DPP-IV) inhibitors (gliptins) [13]. In detail, gliptins are able to rapidly deactivate GLP-1, resulting in the decrease in insulin production, and are a recent addition to the class of oral glucose-lowering drugs used to treat T2D. Several components of this class, including vildagliptin, sitagliptin, saxagliptin, alogliptin, and linagliptin, have already been approved for this indication by the US Food and Drug Administration or by the European Medicines Agency; others are awaiting for approval or still in development. Gliptins can be used as single-agent therapy or combined with other antidiabetic agents (e.g., metformin) when the latter fail to produce or maintain adequate glycemic control [14, 15]. DPP-IVi would indeed induce insulin secretion in a glucose-dependent manner, with minimal risk of hypoglycemia; accordingly, DPP-IVi can produce a significant reduction in HbA1c. Moreover, they are usually well tolerated, with no weight gain or gastrointestinal side effects [15–18]. Interestingly, they also appear to offer added benefits consisting of the epigenetically mediated restoration of normal gene activity in dysfunctional pancreatic islets [19]. A recent study in diabetic CD1 mice also indicates that gliptin therapy can ameliorate T2D-related kidney fibrosis [20], an effect that was mediated by the drug's induction of the expression of miR-29.

These findings prompted us to investigate the circulating miRNA profile of elderly patients with poorly controlled T2D and to identify the changes it undergoes during DPP-IVi therapy with sitagliptin. The plasma levels of three miRNAs (miR-1208, miR-550a-3p, and miR-30c-5p) displayed directionally similar trends in responders and nonresponders during the 15 months of sitagliptin treatment. These miRNAs thus appear to be modulated by sitagliptin, but not correlated with metabolic response. In contrast, three other miRNAs emerged as promising candidates for use as positive (miR-126-3p and miR-223) and negative (miR-378) biomarkers of responses to sitagliptin therapy in this elderly patient population.

## 2. Materials and Methods

### 2.1. Patients

The Ethics Committee of the Tor Vergata University of Rome Medical Center approved this study protocol, and written informed consent was obtained from all patients involved in the study. Patients (males and females) were eligible for enrollment in the study if they met all the following criteria: (1) age > 65 years, (2) a ≥1-year history of T2D, and (3) poor glycemic control (HbA1c levels ranging from 7.5% to 9.0%) on current treatment with maximum-dose metformin. The following exclusion criteria were applied at baseline and during follow-up: insulin therapy, major organ failure (e.g., congestive heart failure and respiratory and/or hepatic insufficiency), positive history for atrial fibrillation or a coronary or cerebrovascular event during the previous 6 months, known neoplastic disease, and/or acute infections.

**Figure 1:**
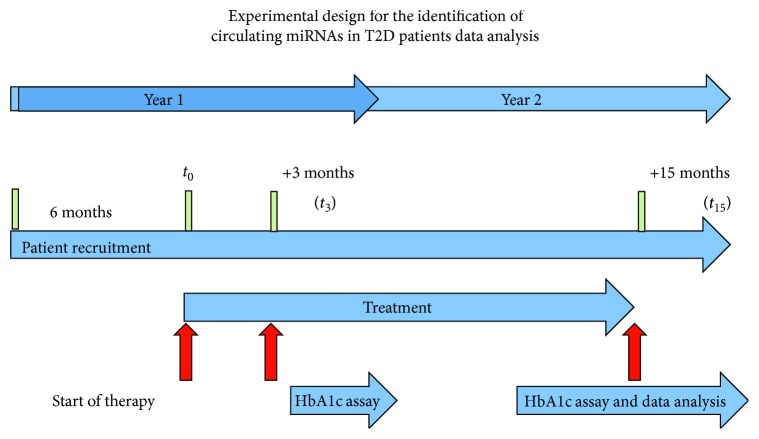
Experimental design for the identification of circulating miRNAs in T2D patients. The duration of the study was 2 years. The blue horizontal arrow indicates the duration of the different phases of the project. The red vertical arrows indicate the main study periods. In year 1, patients were enrolled and started on metformin + sitagliptin (see Materials and Methods for details). After 3 and 15 months of treatment (*t*_3_ and *t*_15_), HbA1c values were reassessed and patients were classified as nonresponders (NR) or responders (R). Plasma miRNA levels at baseline (*t*_0_) and *t*_15_ from groups R and NR were compared as indicated. Comparison of plasma pools: (1) NR-*t*_0_ versus R-*t*_0_, (2) R-*t*_15_ versus R-*t*_0_, (3) NR-*t*_15_ versus NR-*t*_0_, and (4) NR-*t*_15_ versus R-*t*_15_.

**Figure 2:**
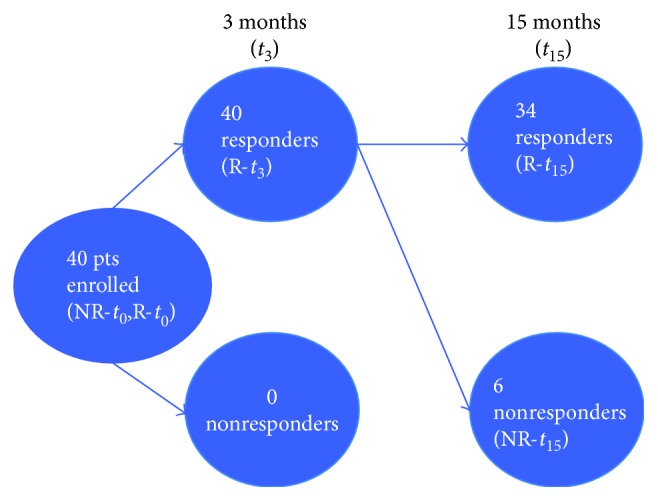
Glycemic control statuses of the patients at baseline and 3 and 15 months after initiation of metformin + sitagliptin. All 40 patients in poor metabolic control were enrolled. HbA1c levels were evaluated after 3 and 15 months from the addition of sitagliptin. On the basis of HbA1c values, patients were divided into responders and nonresponders. All patients showed an initial metabolic response to therapy (*t*_3_), whereas after 15 months (*t*_15_), 34/40 were responders. Based on this information, patients were divided into five groups: (1) *t*_0_ responder samples (R-*t*_0_), (2) *t*_0_ nonresponder samples (NR-*t*_0_), (3) *t*_3_ responder samples (R-*t*_3_), (4) *t*_15_ responder samples (R-*t*_15_), and (5) *t*_15_ nonresponder samples (NR-*t*_15_). miRNA profiling was performed at baseline and after 15 months of sitagliptin addition.

Blood samples for HbA1c measurement and circulating miRNA profiling (details below) were drawn upon enrollment (*t*_0_), and the patients were then started on sitagliptin (100 mg once daily) as an adjunct to their metformin therapy. Glycemic control was assessed 3 and 15 months later (*t*_3_ and *t*_15_, resp.) (as shown in Figure 1). Patients were classified as “responders” (R) if they exhibited good glycemic control at both time points, reflected by an HbA1c level of <7.5% or an HbA1c reduction of >0.5% relative to the level recorded at *t*_0_. Patients who failed to meet these criteria were classified as “nonresponders” (NR). On the basis of these findings, plasma samples were pooled into the following five groups: (1) baseline samples from patients that emerged as responders (R-*t*_0_ pool), (2) baseline samples from nonresponders (NR-*t*_0_ pool), (3) *t*_3_ samples from responders (R-*t*_3_ pool), (4) *t*_15_ samples from responders (R-*t*_15_ pool), and (5) *t*_15_ samples from nonresponders (NR-*t*_15_ pool) (Figure 2). Characteristics of the patients enrolled in the study are reported in Table 1.

### 2.2. Isolation and Profiling of Circulating miRNAs

For miRNA studies, 5 mL of blood was collected from each patient in EDTA-treated tubes. Within 30 minutes of collection, the samples were centrifuged for 10 minutes at 2000 ×g at room temperature (RT), and the plasma thus obtained was divided into 250 *μ*L aliquots and stored at −80°C. To eliminate the risk of bias related to hemolysis [21], all plasma samples were visually assessed and those that were hemolyzed, icteric, or lipemic were excluded from the analysis. We also evaluated the expression of miRNAs susceptible to hemolysis, such as miR-324-3p, miR-454, and miR-652 [21]. Indeed, these miRNAs were not detected in our samples, confirming that none of the samples utilized in the study were hemolyzed.

Plasma samples from all patients in a given group (see above) were thawed on ice and pooled. A miRNA ABC purification kit (Applied Biosystems, Thermo Scientific) was used, according to the manufacturer's instructions. Briefly, 50 *μ*L of each plasma pool was mixed with 100 *μ*L of lysis buffer and centrifuged briefly before the addition of 100 nM of ath-miR-159a (used as a positive external control). Samples were then mixed with freshly prepared magnetic beads (80 × 10^6^) and incubated in a magnetic rack (40 min at 30°C). Bead-hybridized miRNAs were then washed to remove any contaminants. Elution buffer (50 *μ*L) was added, and the sample was incubated for 3 minutes in a ThermoMixer (1200 rpm, 70°C) and placed for 1 minute in a magnetic rack to clear solutions. The supernatants were then transferred into clean tubes and placed on ice.

miRNAs were reverse-transcribed using specific primers according to Applied Biosystems protocols. The cDNAs were preamplified using reagents from Applied Biosystems (Thermo Scientific), and the products were subjected to miRNA expression profiling. The latter was performed by RT-qPCR with Taqman Low-Density Array microfluidic cards (Human miR v3.0, Applied Biosystems), as previously described [22].

### 2.3. miRNA Expression Analysis

Statistical analysis was performed with StatMiner™ software, v. 5.0 (Integromics™) [23]. miRNA expression levels in plasma pools were subjected to global expression normalization, and relative levels were calculated with the comparative threshold cycle (Ct) method. miRNAs with Ct values > 33 were excluded. Differential expression between groups was assessed with the limma test and considered statistically significant when *P* values were <0.05. Heat maps were generated in the R environment (http://www.r-project.org/), using differentially expressed miRNAs as input. The Bray-Curtis and average linkage methods were used to cluster samples (hclust) and generate heat maps (heatmap.2).

## 3. Results and Discussion

All 40 enrolled patients completed 15 months of treatment with metformin + sitagliptin without occurrence of adverse events. After three months of the study treatment, all patients met the predefined criteria for good metabolic control. In contrast, at *t*_15_, only 34/40 (85%) were still in good metabolic control; the remaining six (15%) had HbA1c levels that exceeded 7.5% (*n* = 6). On the basis of these findings, patients were divided into responders (group R), which included the 34 patients in good glycemic control at *t*_3_ and *t*_15_, and nonresponders (group NR, 6/40), whose initial response to the addition of sitagliptin at *t*_3_ was not maintained at *t*_15_.

### 3.1. Circulating miRNA Profiles of Groups R and NR

**Figure 3:**
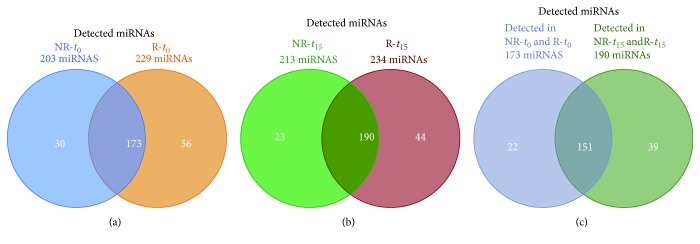
Venn diagram of circulating miRNAs detected in the elderly T2D cohort and in the R and NR subcohorts. Patients were classified as responders (R) or nonresponders (NR) based on their glycemic control status after 15 months of treatment with metformin + sitagliptin (*t*_15_). Venn diagrams show the number of miRNAs detected in NR and R plasma samples collected (a) before the start of combined therapy (baseline, *t*_0_) and (b) at *t*_15_ and (c) at both *t*_0_ and *t*_15_.

We then compared groups R and NR in terms of miRNA expression profiles in their *t*_0_ plasma sample pools. Of the 754 miRNAs analyzed, 203 (27%) were detected in group NR and 229 (30%) in group R (Supplementary Tables 1 and 2, resp.). The reliability of our findings is supported by the fact that 173 miRNAs found in both groups of plasma samples (Figure 3(a)) included several miRNAs previously known to be associated with T2D; specifically, 36% of those were found in the BioM2MetDisease database (http://www.bio-bigdata.com/BioM2MetDisease/browse) and identified in a recent systematic review by He et al. [24] (Table 2). Similar findings emerged when we compared circulating miRNA profiles after 15 months of combined metformin + sitagliptin therapy. Indeed, the number of detectable miRNAs in the NR group (213/754, 28%) was comparable to that in the R group (234/754, 31%) (Supplementary Tables 3 and 4). In addition, the subset of miRNAs found in both *t*_15_ pools (Figure 3(b)) comprised many of those with known links to T2D (39% in BioM2MetDisease and He et al. [24]) (Table 3). Finally, 151 miRNAs were detected in the plasma pools for both groups, before (*t*_0_) and after the addition of sitagliptin (*t*_15_) (Figure 3(c), Supplementary Table 6). Notably, miRNAs described to be linked to T2D in previous studies were detected also in our cohort of elderly T2D patients, further strengthening their potential role as T2D biomarkers.

### 3.2. Circulating miRNAs in Elderly T2D Patients: Differential Expression between Responders and Nonresponders at Baseline

**Figure 4:**
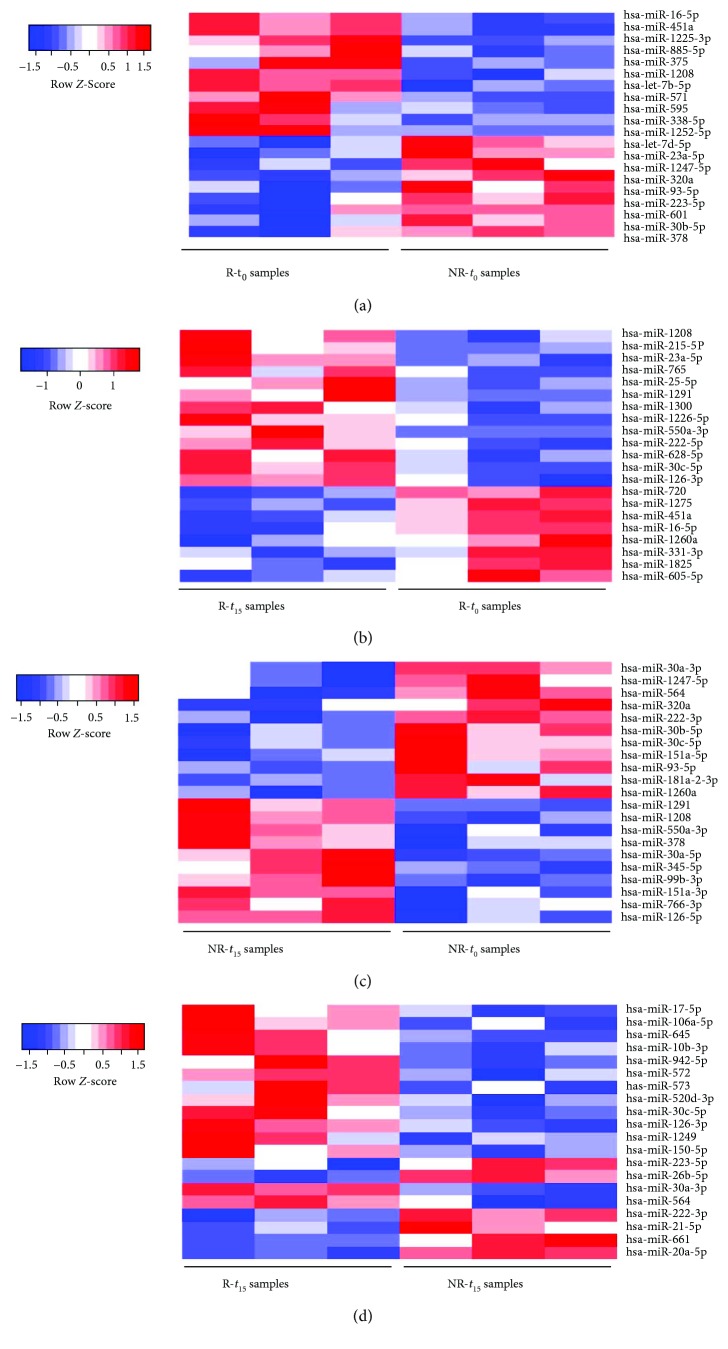
Heat maps showing circulating microRNAs that were differentially expressed in plasma samples from (a) R and NR patients at baseline (*t*_0_), prior to the addition of sitagliptin to the maximum-dose metformin regimen; (b) R at *t*_0_ and after 15 months of sitagliptin (*t*_15_); (c) NR patients at *t*_15_ and *t*_0_; and (d) R and NR patients at *t*_15_.

Our next goal was to identify circulating miRNAs that might be correlated with a positive metabolic response to therapy. To this end, we first compared the plasma levels of the 173 miRNAs found in the *t*_0_ plasma pools from groups R and NR. This analysis revealed 20 miRNAs that were differentially expressed in the two groups (Table 4, Figure 4(a)) and might thus be potentially useful for predicting the response to therapy. As noted in Table 4, plasma levels of eight of these 20 miRNAs are known to be potentially influenced by blood cell contamination [21]. However, this factor is unlikely to have played a role in this analysis, since occurrence of hemolysis was excluded in all plasma samples analyzed.

Expression of 10 miRNAs was significantly lower in plasma from NR-t_0_ versus R-t_0_ (Table 4, Figure 4(a)); these included three miRNAs of particular interest: let-7d, miR-223, and miR-23a, whose expression was reported to be downregulated in T2D patients when compared to healthy individuals [6, 12, 25, 26]. Reduced expression of let-7d, miR-223, and miR-23a in NR patients suggests that these miRNAs may represent potential biomarkers of response to sitagliptin therapy.

Other miRNAs resulted to be significantly more expressed in NR-*t*_0_ plasma samples. These included miR-375, whose high expression has been associated with *β*-cell dysfunction [27, 28]; miR-571, previously reported to be hyperexpressed in plasma samples from T2D patients compared to healthy controls [29]; and miR-378, which was associated with obesity-related insulin resistance [30, 31].

Increasing evidence has shown the involvement of miRNAs in the development of the endocrine pancreas, as well as in the regulation of insulin secretion, insulin signaling, and insulin gene transcription. Indeed, in Dicer-1 conditional knockout mice, loss of miRNAs in *β*-cells causes major defects in glucose homeostasis and in insulin secretion, with a marked reduction in insulin content. Moreover, studies in rodent models of T2D have revealed changes in miRNA expression in *β*-cells [32].

In this context, our data provide evidence that some miRNAs previously reported as regulators of glucose homeostasis in critical tissues (e.g., endocrine pancreas) may also represent circulating biomarkers for disease staging and/or for predicting response to glucose-lowering therapy.

### 3.3. Circulating miRNAs in the Elderly T2D Patients Who Responded to Sitagliptin Treatment

To identify miRNAs potentially modulated by sitagliptin therapy, we compared miRNA expression levels at *t*_0_ and at *t*_15_ in plasma samples from the R group of patients. Twenty-one miRNAs were differentially expressed between the two plasma pools (Table 5, Figure 4(b)). Of note, expression levels of miR-222, previously reported to be hyperexpressed in plasma samples from T2D patients versus healthy controls [33], were reduced at *t*_15_ versus *t*_0_.

Conversely, three miRNAs were found significantly upregulated in the R-*t*_15_ plasma pool (Table 5). These included miR-126-3p, which was reported to be decreased in T2D patients (with or without complications) versus healthy controls [34] and in T2D patients with major cardiovascular events [35]. Moreover, this miRNA has been proposed as a biomarker for the detection of prediabetes and diabetes. miR-30c, another miRNA significantly upregulated in the R-*t*_15_ plasma pool, was recently shown to exert protective effects on diabetic nephropathy [36] and cardiomyopathy [37]. Interestingly, gliptins inhibit the degradation of several peptides and chemokines and reduce tissue inflammation by suppressing macrophage activation and M2 macrophage response. These findings suggest that glucagon-like peptide-1- (GLP-1-) based treatments provide additional benefits beyond glycemic control, including vascular protection and improved bone health [38].

### 3.4. Circulating miRNAs in Elderly T2D Patients Who Did Not Respond to Sitagliptin Treatment

We then compared miRNA expression levels at *t*_0_ and at *t*_15_ in plasma samples from the NR group of patients. As shown in Table 6 and in Figure 4(c), 21 miRNAs were differentially expressed between the two time points, with 5/21 (miR-1208, miR-550a-3p, miR-30c-5p, miR-1260a, and miR-1291) showing similar posttreatment changes to group R (Tables 4 and 5). Consequently, this observation suggests the exclusion of these five miRNAs as possible biomarkers of response to sitagliptin therapy. Nevertheless, the protective effects of miR-30c-5p on diabetic nephropathy and cardiomyopathy [36, 37] underline the need for further investigation on the functional role of miR-30c in elderly T2D patients.

A comparison between the differentially expressed miRNAs of NR versus R at baseline (Table 4) and NR at *t*_15_ versus baseline (Table 6) allowed us to observe that one of the miRNAs that were more abundant in NR plasma than in R plasma at baseline, miR-378, increased during the treatment period, reaching levels in the NR-*t*_15_ plasma pool that were significantly higher than those in the NR-*t*_0_ pool. This result allowed us to propose miR-378 as a negative biomarker candidate for response to sitagliptin therapy.

In this context, our results could open new perspectives providing the basis for further investigations of the reported dysregulated miRNAs as markers of response to gliptins.

### 3.5. Circulating miRNAs in Elderly T2D Patients after 15 Months of Sitagliptin Treatment

To identify miRNAs that might be affected by sitagliptin treatment, we compared circulating miRNA levels in plasma samples at *t*_15_ from the two subgroups. Twenty miRNAs were differentially expressed in plasma from NR versus R patients (Table 7, Figure 4(d)). Ten of the 20 miRNAs were significantly more expressed in the plasma samples from the NR group. Among the upregulated miRNAs, we observed an increased expression of miR-661 and miR-572 in NR-*t*_15,_ in accordance with previous studies performed in T2D patients [26, 39].

miRNAs that were hypoexpressed in the NR-*t*_15_ pool included two of particular interest: miR-126-3p and miR-223. In the previous analysis of group R, miR-126-3p levels at *t*_15_ were higher than those in the *t*_0_ plasma pool, suggesting that this miRNA is a good candidate biomarker of successful metabolic response to therapy. Additionally, our previous comparison of *t*_0_ plasma pools from the two groups revealed substantially lower levels of miR-223 in NR. This miRNA has already been associated with pancreatic islet *β*-cell function and glycemic control, and its expression is reportedly higher in individuals with pre-T2D and normal controls than in T2D patients [40, 41]. In light of these results, miR-223 appears to be a possible positive biomarker for monitoring patients' responsiveness to sitagliptin therapy.

Finally, we checked for a possible correlation between differentially expressed miRNAs, miR-126-3p, miR-223, and miR-378, and the patients' clinical features. We did not observe any statistically significant correlation between miR-126-3p, miR-223, and miR-378 and age, time from T2D diagnosis, body weight, and HbA1c levels.

An increasing body of evidence has linked diabetes to cardiovascular disease, renal failure, neuropathy, and osteoporosis, especially in elderly individuals, with a consequent increase in mortality, morbidity, and socioeconomic costs. Reliable biomarkers to predict drug response in these patients are urgently needed but still lacking. Glucose-lowering drugs, such as DPP-IVi, are known to differentially impact metabolic control and disease-related complications.

Our study is the first to provide a description of the circulating miRNAs that can be used as novel biomarkers for monitoring response to therapy in elderly T2D patients.

## 4. Conclusion

The results we obtained suggest that miR-378, miR-126-3p, and miR-223 represent candidate plasma biomarkers for disease staging and for predicting response to therapy in T2D elderly patients. High circulating levels of miR-378 appear to be a negative predictor of response to sitagliptin in elderly T2D patients. Indeed, miR-378 was more expressed in the NR-*t*_0_ plasma pool than in the R-*t*_0_ pool. In addition, its levels in the NR-*t*_15_ plasma were even higher than those found in the NR-*t*_0_ pool, and this result highlights its possible role as a biomarker of resistance to sitagliptin. In contrast, miR-126-3p and miR-223 seem to be markers of response to the drug. miR-126-3p levels have been reported to be lower in T2D patients than in healthy individuals [34]. Consistently, this miRNA was not differentially expressed in the plasma of R and NR patients at baseline. After 15 months of sitagliptin therapy, however, plasma levels in responders were significantly higher than those found in the NR group, suggesting that the addition of the DPP-IVi may have restored miR-126-3p levels to the range found in healthy subjects. As for miR-223, its expression at baseline was already significantly higher in the R group, and this difference persisted after 15 months of sitagliptin addition. This behavior is consistent with its potential role as a positive predictor of response to the drug.

Further work is needed to validate the role of these miRNAs as biomarkers in T2D patients, since this was a discovery study and the number of NR patients was limited. Interestingly, circulating miRNAs may reflect phenomena occurring at the level of those organs involved in T2D pathophysiology (e.g., endocrine pancreas, liver, and adipose tissue). Therefore, our data provide a snapshot of the circulating miRNAs that deserve further studies.

The translational relevance of our findings is immediate; in fact, data regarding miRNA profiles can produce results of potential impact not only predicting drug response of T2D elderly patients but also helping in selecting patients that could be suitable candidates to this therapy.

## Figures and Tables

**Table 1:** Characteristics of R and NR patients enrolled in the study.

Parameters	Responders (R)			Nonresponders (NR)		
Age (y)	66.62 ± 2.31			67.89 ± 2.24		
Gender, males/females (*n*)	17/17			4/2		
Time since T2D diagnosis (y)	11 ± 2			10 ± 3		
Time points (in months)	*t*_0_	*t*_3_	*t*_15_	*t*_0_	*t*_3_	*t*_15_
Body weight (kg)	72.30 ± 4.92	70.36 ± 4.89	70.43 ± 5.23	79.10 ± 12.19	78.57 ± 13.25	79.33 ± 12.99
HbA1c (%)	7.75 ± 0.38	6.38 ± 0.18	7.10 ± 0.28	7.59 ± 0.16	6.73 ± 0.39	7.81 ± 0.51

Data are means ± SD unless indicated otherwise.

**Table 2:** miRNAs with known links to T2D found in baseline (*t*_0_) plasma pools from groups NR and R.

T2D-linked microRNAs at *t*_0_	
hsa-let-7d	
hsa-miR-103	
hsa-miR-126	
hsa-miR-130b	
hsa-miR-142-3p	
hsa-miR-144	
hsa-miR-145	
hsa-miR-146a	
hsa-miR-155	
hsa-miR-17-5p	
hsa-miR-186	
hsa-miR-191	
hsa-miR-192	
hsa-miR-195	
hsa-miR-197	
hsa-miR-20b	
hsa-miR-21	
hsa-miR-222	
hsa-miR-223	
hsa-miR-23a	
hsa-miR-26a	
hsa-miR-27a	
hsa-miR-29a	
hsa-miR-30d	
hsa-miR-30e	
hsa-miR-342	
hsa-miR-34a	
hsa-miR-375	
hsa-miR-378	
hsa-miR-423-5p	
hsa-miR-451a	
hsa-miR-483-3p	
hsa-miR-486	
hsa-miR-571	
hsa-miR-572	
hsa-miR-593	
hsa-miR-661	
hsa-miR-770-5p	
hsa-miR-9	
hsa-miR-92a	

**Table 3:** miRNAs with known links to T2D found in *t*_15_ plasma pools from groups NR and R.

T2D-linked miRNAs at *t*_15_	
hsa-let-7d	
hsa-miR-103	
hsa-miR-106b	
hsa-miR-122	
hsa-miR-126	
hsa-miR-130b	
hsa-miR-132	
hsa-miR-140	
hsa-miR-140-3p	
hsa-miR-142-3p	
hsa-miR-144	
hsa-miR-145	
hsa-miR-146a	
hsa-miR-155	
hsa-miR-17-5p	
hsa-miR-181a	
hsa-miR-186	
hsa-miR-18a	
hsa-miR-191	
hsa-miR-192	
hsa-miR-195	
hsa-miR-197	
hsa-miR-20b	
hsa-miR-21	
hsa-miR-221	
hsa-miR-222	
hsa-miR-223	
hsa-miR-23a	
hsa-miR-24	
hsa-miR-26a	
hsa-miR-27a	
hsa-miR-28-3p	
hsa-miR-30d	
hsa-miR-30e	
hsa-miR-320	
hsa-miR-342	
hsa-miR-34a	
hsa-miR-375	
hsa-miR-378	
hsa-miR-423-5p	
hsa-miR-451a	
hsa-miR-483-3p	
hsa-miR-486	
hsa-miR-571	
hsa-miR-572	
hsa-miR-593	
hsa-miR-661	
hsa-miR-770-5p	
hsa-miR-92a	
hsa-miR-96	

**Table 4:** miRNAs that were differentially expressed in *t*_0_ plasma pools from groups NR and R.

Regulation	miRNA	Linear fold change	*P* value
Upregulated in NR-*t*_0_	hsa-miR-1208	2.05	1.84*E* − 02
	hsa-miR-1225-3p	2.27	1.20*E* − 02
	hsa-miR-1252-5p	901.88	4.52*E* − 02
	hsa-miR-338-5p	7.56	4.22*E* − 02
	hsa-miR-375	68.83	4.35*E* − 02
	hsa-miR-378^∗^	15.26	3.81*E* − 02
	hsa-miR-571	2.39	1.32*E* − 02
	hsa-miR-595	540.84	4.66*E* − 02
	hsa-miR-601^∗^	14179.56	4.97*E* − 02
	hsa-miR-885-5p	4.58	3.00*E* − 02

Downregulated in NR-*t*_0_	hsa-let-7b-5p^∗^	0.18	1.53*E* − 03
	hsa-let-7d-5p	0.19	1.11*E* − 02
	hsa-miR-1247-5p	0.20	9.91*E* − 03
	hsa-miR-16-5p^∗^	0.21	1.51*E* − 03
	hsa-miR-223-5p	0.32	1.83*E* − 02
	hsa-miR-23a-5p^∗^	0.20	8.79*E* − 03
	hsa-miR-30b-5p^∗^	0.36	2.74*E* − 02
	hsa-miR-320a	0.23	5.22*E* − 03
	hsa-miR-451a^∗^	0.31	3.88*E* − 03
	hsa-miR-93-5p^∗^	0.09	2.03*E* − 02

^∗^Hemolysis-susceptible miRNAs as reported in Kirschner et al. [21].

**Table 5:** miRNAs that were differentially expressed in *t*_0_ and *t*_15_ plasma pools from group R.

Regulation	miRNA	Linear fold change	*P* value
Upregulated in R-*t*_15_	hsa-miR-126-3p	3.30	2.05*E* − 02
	hsa-miR-30c-5p	3.44	4.71*E* − 03
	hsa-miR-331-3p^∗^	163.08	3.12*E* − 02

Downregulated in R-*t*_15_	hsa-miR-1208	0.53	3.55*E* − 02
	hsa-miR-1226-5p	0.42	2.78*E* − 02
	hsa-miR-1260a	0.35	4.07*E* − 02
	hsa-miR-1275	0.24	1.93*E* − 03
	hsa-miR-1291	0.15	3.98*E* − 02
	hsa-miR-1300	0.29	2.08*E* − 02
	hsa-miR-16-5p^∗^	0.27	2.23*E* − 02
	hsa-miR-1825	0.50	4.13*E* − 02
	hsa-miR-215-5p	0.05	3.76*E* − 02
	hsa-miR-222-5p	0.26	3.66*E* − 02
	hsa-miR-23a-5p^∗^	0.18	4.28*E* − 03
	hsa-miR-25-5p^∗^	0.03	3.26*E* − 02
	hsa-miR-451a^∗^	0.35	9.49*E* − 03
	hsa-miR-550a-3p	0.34	1.27*E* − 02
	hsa-miR-605-5p	0.19	1.34*E* − 02
	hsa-miR-628-5p	0.00	3.07*E* − 02
	hsa-miR-720	0.41	5.80*E* − 03
	hsa-miR-765	0.01	4.16*E* − 02

^∗^Hemolysis-susceptible miRNAs as reported in Kirschner et al. [21].

**Table 6:** miRNAs that were differentially expressed in *t*_0_ and *t*_15_ plasma pools from group NR.

Regulation	miRNA	Linear fold change	*P* value
Upregulated in NR-*t*_15_	hsa-miR-1247-5p	4.57	2.50*E* − 02
	hsa-miR-126-5p	1.62	4.72*E* − 02
	hsa-miR-151a-3p	2.50	1.87*E* − 02
	hsa-miR-151a-5p	3.73	9.29*E* − 03
	hsa-miR-181a-2-3p	134.20	1.75*E* − 02
	hsa-miR-222-3p	1.85	1.94*E* − 02
	hsa-miR-30a-3p^∗^	1.70	4.95*E* − 02
	hsa-miR-30a-5p	1.59	4.80*E* − 02
	hsa-miR-30b-5p	2.18	2.63*E* − 02
	hsa-miR-30c-5p	2.08	3.87*E* − 02
	hsa-miR-320a	2.95	3.40*E* − 02
	hsa-miR-378^∗^	2.05	4.62*E* − 02
	hsa-miR-564	3.39	1.41*E* − 02
	hsa-miR-766-3p	3.46	4.62*E* − 02
	hsa-miR-93-5p^∗^	6.81	2.63*E* − 02

Downregulated in NR-*t*_15_	hsa-miR-1208	0.23	3.70*E* − 03
	hsa-miR-1260a	0.37	8.75*E* − 03
	hsa-miR-1291	0.28	1.11*E* − 02
	hsa-miR-345-5p	0.15	9.84*E* − 03
	hsa-miR-550a-3p	0.48	4.08*E* − 02
	hsa-miR-99b-3p^∗^	0.29	7.73*E* − 03

^∗^Hemolysis-susceptible miRNAs as reported in Kirschner et al. [21].

**Table 7:** miRNAs that were differentially expressed in the *t*_15_ plasma pools from groups NR and R.

Regulation	miRNA	Linear fold change	*P* value
Upregulated in NR-*t*_15_	hsa-miR-10b-3p	2.49	3.39*E* − 02
	hsa-miR-1249	4.17	4.27*E* − 02
	hsa-miR-30a-3p^∗^	3.84	1.62*E* − 03
	hsa-miR-520d-3p	6.78	1.79*E* − 02
	hsa-miR-564	2.86	2.19*E* − 02
	hsa-miR-572	2.15	1.81*E* − 02
	hsa-miR-573	7.99	4.10*E* − 02
	hsa-miR-645	2.85	1.90*E* − 02
	hsa-miR-661	6.57	3.68*E* − 03
	hsa-miR-942-5p	2.63	1.70*E* − 02

Downregulated in NR-*t*_15_	hsa-miR-106a-5p^∗^	0.41	2.68*E* − 02
	hsa-miR-126-3p	0.42	1.13*E* − 02
	hsa-miR-150-5p	0.49	4.21*E* − 02
	hsa-miR-17-5p^∗^	0.43	3.28*E* − 02
	hsa-miR-20a-5p^∗^	0.18	3.61*E* − 04
	hsa-miR-21-5p^∗^	0.17	1.59*E* − 02
	hsa-miR-222-3p	0.35	5.14*E* − 03
	hsa-miR-223-5p	0.54	4.95*E* − 02
	hsa-miR-26b-5p^∗^	0.41	4.04*E* − 03
	hsa-miR-30c-5p	0.45	2.62*E* − 02

^∗^Hemolysis-susceptible miRNAs as reported in Kirschner et al. [21].

## Supplementary Materials

Supplementary Materials Supplementary Tables 1–5 list the detected microRNAs in the plasma samples of interest (NR-*t*_0_, R-*t*_0_, NR-*t*_15_, and R-*t*_15_).
