# Supplementary material for: Circulating MicroRNAs in Elderly Type 2 Diabetic Patients
Source: Int J Endocrinol. 2018 Apr 10;2018:6872635. doi: 10.1155/2018/6872635 (PMC5914089; doi:10.1155/2018/6872635)
Supplement: Supplementary Materials — Supplementary Tables 1–5 list the detected microRNAs in the plasma samples of interest (NR-t 0, R-t 0, NR-t 15, and R-t 15). [file 6872635.f1.docx]

**Supplementary Table 1.** **MiRNAs detected in NR-t_0_ plasma samples**

| microRNA |
| --- |
| hsa-let-7b-5p |
| hsa-let-7d-5p |
| hsa-let-7e-5p |
| hsa-miR-103a-3p |
| hsa-miR-106a-5p |
| hsa-miR-106b-5p |
| hsa-miR-10a-5p |
| hsa-miR-10b-3p |
| hsa-miR-1180-3p |
| hsa-miR-1183 |
| hsa-miR-1208 |
| hsa-miR-122-5p |
| hsa-miR-1225-3p |
| hsa-miR-1226-5p |
| hsa-miR-1228-5p |
| hsa-miR-1233-3p |
| hsa-miR-1247-5p |
| hsa-miR-1249 |
| hsa-miR-1252-5p |
| hsa-miR-1253 |
| hsa-miR-1254 |
| hsa-miR-126-3p |
| hsa-miR-126-5p |
| hsa-miR-1260a |
| hsa-miR-1262 |
| hsa-miR-127-5p |
| hsa-miR-1274A |
| hsa-miR-1274B |
| hsa-miR-1275 |
| hsa-miR-1276 |
| hsa-miR-1286 |
| hsa-miR-129-5p |
| hsa-miR-1290 |
| hsa-miR-1291 |
| hsa-miR-1300 |
| hsa-miR-1301-3p |
| hsa-miR-1305 |
| hsa-miR-130b-3p |
| hsa-miR-133a-3p |
| hsa-miR-139-5p |
| hsa-miR-140-5p |
| hsa-miR-142-3p |
| hsa-miR-144-3p |
| hsa-miR-144-5p |
| hsa-miR-145-3p |
| hsa-miR-146a-5p |
| hsa-miR-146b-5p |
| hsa-miR-150-5p |
| hsa-miR-151a-3p |
| hsa-miR-151a-5p |
| hsa-miR-155-5p |
| hsa-miR-15b-3p |
| hsa-miR-15b-5p |
| hsa-miR-16-5p |
| hsa-miR-17-5p |
| hsa-miR-181a-2-3p |
| hsa-miR-181a-3p |
| hsa-miR-1825 |
| hsa-miR-183-3p |
| hsa-miR-185-3p |
| hsa-miR-185-5p |
| hsa-miR-186-5p |
| hsa-miR-187-3p |
| hsa-miR-18a-3p |
| hsa-miR-190a-5p |
| hsa-miR-190b |
| hsa-miR-190b |
| hsa-miR-191-5p |
| hsa-miR-192-5p |
| hsa-miR-193a-5p |
| hsa-miR-194-5p |
| hsa-miR-195-5p |
| hsa-miR-197-3p |
| hsa-miR-199b-3p |
| hsa-miR-19a-3p |
| hsa-miR-19b-1-5p |
| hsa-miR-19b-3p |
| hsa-miR-206 |
| hsa-miR-20a-5p |
| hsa-miR-20b-5p |
| hsa-miR-21-3p |
| hsa-miR-21-5p |
| hsa-miR-212-3p |
| hsa-miR-215-5p |
| hsa-miR-22-5p |
| hsa-miR-222-3p |
| hsa-miR-222-5p |
| hsa-miR-223-3p |
| hsa-miR-223-5p |
| hsa-miR-23a-5p |
| hsa-miR-24-2-5p |
| hsa-miR-25-3p |
| hsa-miR-26a-5p |
| hsa-miR-26b |
| hsa-miR-26b-3p |
| hsa-miR-26b-5p |
| hsa-miR-27a-3p |
| hsa-miR-29a-5p |
| hsa-miR-30a-3p |
| hsa-miR-30a-5p |
| hsa-miR-30b-5p |
| hsa-miR-30c-5p |
| hsa-miR-30d-3p |
| hsa-miR-30d-5p |
| hsa-miR-30e-3p |
| hsa-miR-320a |
| hsa-miR-320b |
| hsa-miR-323a-3p |
| hsa-miR-328-3p |
| hsa-miR-335-3p |
| hsa-miR-335-5p |
| hsa-miR-338-5p |
| hsa-miR-340-3p |
| hsa-miR-342-3p |
| hsa-miR-345-5p |
| hsa-miR-346 |
| hsa-miR-34a-3p |
| hsa-miR-34b-3p |
| hsa-miR-363-3p |
| hsa-miR-374b-3p |
| hsa-miR-375 |
| hsa-miR-378 |
| hsa-miR-378a-5p |
| hsa-miR-380-5p |
| hsa-miR-411-3p |
| hsa-miR-423-5p |
| hsa-miR-425-3p |
| hsa-miR-425-5p |
| hsa-miR-432-3p |
| hsa-miR-451a |
| hsa-miR-483-3p |
| hsa-miR-483-5p |
| hsa-miR-484 |
| hsa-miR-485-3p |
| hsa-miR-486-5p |
| hsa-miR-488-5p |
| hsa-miR-495-3p |
| hsa-miR-505-5p |
| hsa-miR-512-3p |
| hsa-miR-516a-3p |
| hsa-miR-518d-3p |
| hsa-miR-518e-5p |
| hsa-miR-518f-5p |
| hsa-miR-520c-3p |
| hsa-miR-520d-3p |
| hsa-miR-520d-5p |
| hsa-miR-548c-3p |
| hsa-miR-550a-3p |
| hsa-miR-554 |
| hsa-miR-564 |
| hsa-miR-566 |
| hsa-miR-571 |
| hsa-miR-572 |
| hsa-miR-573 |
| hsa-miR-574-3p |
| hsa-miR-583 |
| hsa-miR-584-5p |
| hsa-miR-593-5p |
| hsa-miR-595 |
| hsa-miR-597-5p |
| hsa-miR-600 |
| hsa-miR-601 |
| hsa-miR-603 |
| hsa-miR-605-5p |
| hsa-miR-615-3p |
| hsa-miR-616-5p |
| hsa-miR-623 |
| hsa-miR-624-5p |
| hsa-miR-625-3p |
| hsa-miR-626 |
| hsa-miR-628-3p |
| hsa-miR-628-5p |
| hsa-miR-636 |
| hsa-miR-638 |
| hsa-miR-639 |
| hsa-miR-659-3p |
| hsa-miR-660-5p |
| hsa-miR-661 |
| hsa-miR-663b |
| hsa-miR-664a-3p |
| hsa-miR-675-5p |
| hsa-miR-7-1-3p |
| hsa-miR-7-2-3p |
| hsa-miR-708-5p |
| hsa-miR-720 |
| hsa-miR-744-3p |
| hsa-miR-758-3p |
| hsa-miR-765 |
| hsa-miR-766-3p |
| hsa-miR-769-3p |
| hsa-miR-769-5p |
| hsa-miR-770-5p |
| hsa-miR-874-3p |
| hsa-miR-875-5p |
| hsa-miR-885-5p |
| hsa-miR-888-3p |
| hsa-miR-9-3p |
| hsa-miR-921 |
| hsa-miR-92a-3p |
| hsa-miR-93-3p |
| hsa-miR-93-5p |
| hsa-miR-943 |
| hsa-miR-99b-3p |

**Supplementary Table 2.** **MiRNAs detected in the R-t_0_ plasma sample pool**

| microRNA |
| --- |
| hsa-let-7a-5p |
| hsa-let-7b-5p |
| hsa-let-7d-5p |
| hsa-let-7e-5p |
| hsa-let-7g-5p |
| hsa-miR-103a-3p |
| hsa-miR-106a-5p |
| hsa-miR-106b-3p |
| hsa-miR-10a-5p |
| hsa-miR-10b-3p |
| hsa-miR-1180-3p |
| hsa-miR-1183 |
| hsa-miR-1205 |
| hsa-miR-1208 |
| hsa-miR-122-5p |
| hsa-miR-1225-3p |
| hsa-miR-1226-5p |
| hsa-miR-1227-3p |
| hsa-miR-1228-5p |
| hsa-miR-1233-3p |
| hsa-miR-1247-5p |
| hsa-miR-1249 |
| hsa-miR-1252-5p |
| hsa-miR-1253 |
| hsa-miR-1257 |
| hsa-miR-125b-5p |
| hsa-miR-126-3p |
| hsa-miR-126-5p |
| hsa-miR-1260a |
| hsa-miR-1262 |
| hsa-miR-1269a |
| hsa-miR-127-3p |
| hsa-miR-1270 |
| hsa-miR-1274A |
| hsa-miR-1274B |
| hsa-miR-1275 |
| hsa-miR-1276 |
| hsa-miR-1285-3p |
| hsa-miR-1286 |
| hsa-miR-1290 |
| hsa-miR-1291 |
| hsa-miR-1298-5p |
| hsa-miR-1300 |
| hsa-miR-1303 |
| hsa-miR-1305 |
| hsa-miR-130a-3p |
| hsa-miR-130b-3p |
| hsa-miR-136-3p |
| hsa-miR-139-5p |
| hsa-miR-142-3p |
| hsa-miR-144-3p |
| hsa-miR-144-5p |
| hsa-miR-145-3p |
| hsa-miR-145-5p |
| hsa-miR-146a-5p |
| hsa-miR-146b-5p |
| hsa-miR-148a-3p |
| hsa-miR-148b-3p |
| hsa-miR-150-5p |
| hsa-miR-151a-3p |
| hsa-miR-151a-5p |
| hsa-miR-155-5p |
| hsa-miR-15a-3p |
| hsa-miR-15b-3p |
| hsa-miR-15b-5p |
| hsa-miR-16-5p |
| hsa-miR-17-5p |
| hsa-miR-181a-2-3p |
| hsa-miR-1825 |
| hsa-miR-183-3p |
| hsa-miR-185-3p |
| hsa-miR-185-5p |
| hsa-miR-186-5p |
| hsa-miR-190b |
| hsa-miR-190b |
| hsa-miR-191-3p |
| hsa-miR-191-5p |
| hsa-miR-192-5p |
| hsa-miR-194-3p |
| hsa-miR-195-5p |
| hsa-miR-197-3p |
| hsa-miR-199b-3p |
| hsa-miR-19a-3p |
| hsa-miR-19b-1-5p |
| hsa-miR-19b-3p |
| hsa-miR-206 |
| hsa-miR-20a-3p |
| hsa-miR-20a-5p |
| hsa-miR-20b-5p |
| hsa-miR-21-3p |
| hsa-miR-21-5p |
| hsa-miR-212-3p |
| hsa-miR-215-5p |
| hsa-miR-22-5p |
| hsa-miR-221-3p |
| hsa-miR-222-3p |
| hsa-miR-222-5p |
| hsa-miR-223-3p |
| hsa-miR-223-5p |
| hsa-miR-23a-5p |
| hsa-miR-25-3p |
| hsa-miR-25-5p |
| hsa-miR-26a-5p |
| hsa-miR-26b |
| hsa-miR-26b-3p |
| hsa-miR-26b-5p |
| hsa-miR-27a-3p |
| hsa-miR-28-3p |
| hsa-miR-29a-3p |
| hsa-miR-29a-5p |
| hsa-miR-29c-5p |
| hsa-miR-30a-3p |
| hsa-miR-30a-5p |
| hsa-miR-30b-5p |
| hsa-miR-30c-5p |
| hsa-miR-30d-3p |
| hsa-miR-30d-5p |
| hsa-miR-30e-3p |
| hsa-miR-320a |
| hsa-miR-320b |
| hsa-miR-323a-3p |
| hsa-miR-328-3p |
| hsa-miR-331-3p |
| hsa-miR-335-3p |
| hsa-miR-335-5p |
| hsa-miR-337-3p |
| hsa-miR-338-5p |
| hsa-miR-33a-5p |
| hsa-miR-33a-5p |
| hsa-miR-340-3p |
| hsa-miR-342-3p |
| hsa-miR-346 |
| hsa-miR-34a-3p |
| hsa-miR-34b-3p |
| hsa-miR-361-5p |
| hsa-miR-363-3p |
| hsa-miR-365b-3p |
| hsa-miR-374b-3p |
| hsa-miR-374b-5p |
| hsa-miR-375 |
| hsa-miR-376a-3p |
| hsa-miR-378 |
| hsa-miR-409-3p |
| hsa-miR-411-3p |
| hsa-miR-423-5p |
| hsa-miR-425-3p |
| hsa-miR-425-5p |
| hsa-miR-432-3p |
| hsa-miR-432-5p |
| hsa-miR-451a |
| hsa-miR-483-3p |
| hsa-miR-483-5p |
| hsa-miR-484 |
| hsa-miR-485-3p |
| hsa-miR-486-3p |
| hsa-miR-486-5p |
| hsa-miR-488-5p |
| hsa-miR-489-3p |
| hsa-miR-495-3p |
| hsa-miR-497-5p |
| hsa-miR-505-5p |
| hsa-miR-516a-3p |
| hsa-miR-518d-3p |
| hsa-miR-518f-3p |
| hsa-miR-518f-5p |
| hsa-miR-519b-3p |
| hsa-miR-520c-3p |
| hsa-miR-520d-3p |
| hsa-miR-520d-5p |
| hsa-miR-543 |
| hsa-miR-548c-3p |
| hsa-miR-550a-3p |
| hsa-miR-550a-5p |
| hsa-miR-555 |
| hsa-miR-564 |
| hsa-miR-566 |
| hsa-miR-571 |
| hsa-miR-572 |
| hsa-miR-573 |
| hsa-miR-574-3p |
| hsa-miR-578 |
| hsa-miR-584-5p |
| hsa-miR-593-5p |
| hsa-miR-595 |
| hsa-miR-597-5p |
| hsa-miR-600 |
| hsa-miR-601 |
| hsa-miR-603 |
| hsa-miR-605-5p |
| hsa-miR-616-5p |
| hsa-miR-623 |
| hsa-miR-624-5p |
| hsa-miR-625-3p |
| hsa-miR-628-3p |
| hsa-miR-628-5p |
| hsa-miR-629-3p |
| hsa-miR-630 |
| hsa-miR-636 |
| hsa-miR-638 |
| hsa-miR-639 |
| hsa-miR-652-3p |
| hsa-miR-659-3p |
| hsa-miR-660-5p |
| hsa-miR-661 |
| hsa-miR-664a-3p |
| hsa-miR-671-3p |
| hsa-miR-675-5p |
| hsa-miR-7-1-3p |
| hsa-miR-7-2-3p |
| hsa-miR-708-5p |
| hsa-miR-720 |
| hsa-miR-765 |
| hsa-miR-766-3p |
| hsa-miR-769-5p |
| hsa-miR-770-5p |
| hsa-miR-875-5p |
| hsa-miR-885-5p |
| hsa-miR-888-3p |
| hsa-miR-892b |
| hsa-miR-9-3p |
| hsa-miR-921 |
| hsa-miR-92a-3p |
| hsa-miR-93-3p |
| hsa-miR-93-5p |
| hsa-miR-939-5p |
| hsa-miR-942-5p |
| hsa-miR-96-3p |
| hsa-miR-99b-3p |
| hsa-miR-99b-5p |

**Supplementary Table 3. MiRNAs detected in the NR-t_15_ plasma pool**

| microRNAs |
| --- |
| hsa-let-7b-5p |
| hsa-let-7c-5p |
| hsa-let-7d-5p |
| hsa-let-7e-5p |
| hsa-miR-103a-3p |
| hsa-miR-106a-5p |
| hsa-miR-106b-3p |
| hsa-miR-106b-5p |
| hsa-miR-10b-3p |
| hsa-miR-1180-3p |
| hsa-miR-1183 |
| hsa-miR-1208 |
| hsa-miR-122-5p |
| hsa-miR-1225-3p |
| hsa-miR-1226-5p |
| hsa-miR-1228-5p |
| hsa-miR-1233-3p |
| hsa-miR-1243 |
| hsa-miR-1244 |
| hsa-miR-1247-5p |
| hsa-miR-1249 |
| hsa-miR-1252-5p |
| hsa-miR-1253 |
| hsa-miR-1254 |
| hsa-miR-125a-5p |
| hsa-miR-126-3p |
| hsa-miR-126-5p |
| hsa-miR-1260a |
| hsa-miR-1274A |
| hsa-miR-1274B |
| hsa-miR-1275 |
| hsa-miR-1276 |
| hsa-miR-128-3p |
| hsa-miR-1286 |
| hsa-miR-1290 |
| hsa-miR-1291 |
| hsa-miR-1300 |
| hsa-miR-1305 |
| hsa-miR-130b-5p |
| hsa-miR-132-3p |
| hsa-miR-136-3p |
| hsa-miR-139-3p |
| hsa-miR-139-5p |
| hsa-miR-140-3p |
| hsa-miR-142-3p |
| hsa-miR-144-3p |
| hsa-miR-144-5p |
| hsa-miR-145-3p |
| hsa-miR-145-5p |
| hsa-miR-146a-5p |
| hsa-miR-146b-5p |
| hsa-miR-148a-3p |
| hsa-miR-148b-5p |
| hsa-miR-150-5p |
| hsa-miR-151a-3p |
| hsa-miR-151a-5p |
| hsa-miR-152-3p |
| hsa-miR-155-5p |
| hsa-miR-16-5p |
| hsa-miR-17-5p |
| hsa-miR-181a-2-3p |
| hsa-miR-1825 |
| hsa-miR-183-3p |
| hsa-miR-185-3p |
| hsa-miR-185-5p |
| hsa-miR-186-5p |
| hsa-miR-18a-3p |
| hsa-miR-190b |
| hsa-miR-191-3p |
| hsa-miR-191-5p |
| hsa-miR-192-5p |
| hsa-miR-195-5p |
| hsa-miR-197-3p |
| hsa-miR-199b-3p |
| hsa-miR-19a-3p |
| hsa-miR-19b-1-5p |
| hsa-miR-19b-3p |
| hsa-miR-200a-3p |
| hsa-miR-206 |
| hsa-miR-20a-3p |
| hsa-miR-20a-5p |
| hsa-miR-20b-5p |
| hsa-miR-21-3p |
| hsa-miR-21-5p |
| hsa-miR-212-3p |
| hsa-miR-214-5p |
| hsa-miR-22-5p |
| hsa-miR-221-3p |
| hsa-miR-222-3p |
| hsa-miR-222-5p |
| hsa-miR-223-3p |
| hsa-miR-223-5p |
| hsa-miR-23a-5p |
| hsa-miR-24-2-5p |
| hsa-miR-25-3p |
| hsa-miR-26a-1-3p |
| hsa-miR-26a-5p |
| hsa-miR-26b |
| hsa-miR-26b-3p |
| hsa-miR-26b-5p |
| hsa-miR-27a-3p |
| hsa-miR-27b-3p |
| hsa-miR-28-3p |
| hsa-miR-302c-5p |
| hsa-miR-30a-3p |
| hsa-miR-30a-5p |
| hsa-miR-30b-5p |
| hsa-miR-30c-5p |
| hsa-miR-30d-3p |
| hsa-miR-30d-5p |
| hsa-miR-30e-3p |
| hsa-miR-320a |
| hsa-miR-320b |
| hsa-miR-323a-3p |
| hsa-miR-328-3p |
| hsa-miR-331-3p |
| hsa-miR-335-3p |
| hsa-miR-335-5p |
| hsa-miR-337-3p |
| hsa-miR-338-5p |
| hsa-miR-33a-5p |
| hsa-miR-342-3p |
| hsa-miR-345-5p |
| hsa-miR-346 |
| hsa-miR-34a-3p |
| hsa-miR-34b-3p |
| hsa-miR-370-3p |
| hsa-miR-374a-5p |
| hsa-miR-374b-3p |
| hsa-miR-374b-5p |
| hsa-miR-375 |
| hsa-miR-378 |
| hsa-miR-378a-5p |
| hsa-miR-380-5p |
| hsa-miR-409-3p |
| hsa-miR-411-3p |
| hsa-miR-423-5p |
| hsa-miR-425-3p |
| hsa-miR-425-5p |
| hsa-miR-432-3p |
| hsa-miR-451a |
| hsa-miR-454-3p |
| hsa-miR-483-3p |
| hsa-miR-483-5p |
| hsa-miR-484 |
| hsa-miR-485-3p |
| hsa-miR-486-5p |
| hsa-miR-488-5p |
| hsa-miR-489-3p |
| hsa-miR-495-3p |
| hsa-miR-505-5p |
| hsa-miR-516a-3p |
| hsa-miR-518a-3p |
| hsa-miR-518b |
| hsa-miR-518d-3p |
| hsa-miR-520c-3p |
| hsa-miR-520d-3p |
| hsa-miR-520d-5p |
| hsa-miR-539-5p |
| hsa-miR-543 |
| hsa-miR-548a-3p |
| hsa-miR-550a-3p |
| hsa-miR-559 |
| hsa-miR-564 |
| hsa-miR-566 |
| hsa-miR-571 |
| hsa-miR-572 |
| hsa-miR-573 |
| hsa-miR-574-3p |
| hsa-miR-583 |
| hsa-miR-584-5p |
| hsa-miR-593-5p |
| hsa-miR-595 |
| hsa-miR-597-5p |
| hsa-miR-600 |
| hsa-miR-601 |
| hsa-miR-603 |
| hsa-miR-605-5p |
| hsa-miR-623 |
| hsa-miR-624-5p |
| hsa-miR-625-3p |
| hsa-miR-628-3p |
| hsa-miR-629-3p |
| hsa-miR-633 |
| hsa-miR-636 |
| hsa-miR-638 |
| hsa-miR-639 |
| hsa-miR-645 |
| hsa-miR-648 |
| hsa-miR-659-3p |
| hsa-miR-660-5p |
| hsa-miR-661 |
| hsa-miR-664a-3p |
| hsa-miR-668-3p |
| hsa-miR-675-5p |
| hsa-miR-7-1-3p |
| hsa-miR-708-5p |
| hsa-miR-720 |
| hsa-miR-744-3p |
| hsa-miR-766-3p |
| hsa-miR-769-5p |
| hsa-miR-770-5p |
| hsa-miR-875-5p |
| hsa-miR-885-5p |
| hsa-miR-888-3p |
| hsa-miR-9-3p |
| hsa-miR-92a-3p |
| hsa-miR-93-3p |
| hsa-miR-93-5p |
| hsa-miR-942-5p |
| hsa-miR-96-3p |
| hsa-miR-99b-3p |
| hsa-miR-99b-5p |

**Supplementary Table 4.** **MiRNAs detected in the R-t_15_ plasma sample pool**

| microRNAs |
| --- |
| hsa-let-7b-5p |
| hsa-let-7c-5p |
| hsa-let-7d-5p |
| hsa-let-7e-5p |
| hsa-let-7g-5p |
| hsa-miR-103a-3p |
| hsa-miR-106a-5p |
| hsa-miR-106b-3p |
| hsa-miR-106b-5p |
| hsa-miR-10a-5p |
| hsa-miR-10b-3p |
| hsa-miR-1180-3p |
| hsa-miR-1183 |
| hsa-miR-1204 |
| hsa-miR-1208 |
| hsa-miR-122-5p |
| hsa-miR-1225-3p |
| hsa-miR-1226-5p |
| hsa-miR-1227-3p |
| hsa-miR-1233-3p |
| hsa-miR-1247-5p |
| hsa-miR-1249 |
| hsa-miR-1252-5p |
| hsa-miR-1253 |
| hsa-miR-1254 |
| hsa-miR-1257 |
| hsa-miR-125a-5p |
| hsa-miR-125b-5p |
| hsa-miR-126-3p |
| hsa-miR-126-5p |
| hsa-miR-1260a |
| hsa-miR-1262 |
| hsa-miR-127-3p |
| hsa-miR-1274A |
| hsa-miR-1274B |
| hsa-miR-1275 |
| hsa-miR-1276 |
| hsa-miR-1285-3p |
| hsa-miR-1286 |
| hsa-miR-1290 |
| hsa-miR-1291 |
| hsa-miR-1298-5p |
| hsa-miR-1300 |
| hsa-miR-1304-5p |
| hsa-miR-1305 |
| hsa-miR-130a-3p |
| hsa-miR-130b-5p |
| hsa-miR-132-3p |
| hsa-miR-133a-3p |
| hsa-miR-136-3p |
| hsa-miR-139-3p |
| hsa-miR-139-5p |
| hsa-miR-140-3p |
| hsa-miR-140-5p |
| hsa-miR-142-3p |
| hsa-miR-144-3p |
| hsa-miR-144-5p |
| hsa-miR-145-5p |
| hsa-miR-146a-5p |
| hsa-miR-146b-5p |
| hsa-miR-148a-3p |
| hsa-miR-148b-3p |
| hsa-miR-148b-5p |
| hsa-miR-150-5p |
| hsa-miR-151a-3p |
| hsa-miR-151a-5p |
| hsa-miR-154-3p |
| hsa-miR-155-5p |
| hsa-miR-15b-3p |
| hsa-miR-15b-5p |
| hsa-miR-16-5p |
| hsa-miR-17-5p |
| hsa-miR-181a-2-3p |
| hsa-miR-181a-3p |
| hsa-miR-1825 |
| hsa-miR-183-3p |
| hsa-miR-185-5p |
| hsa-miR-186-5p |
| hsa-miR-18a-3p |
| hsa-miR-190b |
| hsa-miR-191-3p |
| hsa-miR-191-5p |
| hsa-miR-192-5p |
| hsa-miR-195-5p |
| hsa-miR-197-3p |
| hsa-miR-199b-3p |
| hsa-miR-19a-3p |
| hsa-miR-19b-1-5p |
| hsa-miR-19b-3p |
| hsa-miR-200b-3p |
| hsa-miR-20a-3p |
| hsa-miR-20a-5p |
| hsa-miR-20b-5p |
| hsa-miR-21-3p |
| hsa-miR-21-5p |
| hsa-miR-210-3p |
| hsa-miR-212-3p |
| hsa-miR-215-5p |
| hsa-miR-22-3p |
| hsa-miR-22-5p |
| hsa-miR-221-3p |
| hsa-miR-222-3p |
| hsa-miR-222-5p |
| hsa-miR-223-3p |
| hsa-miR-223-5p |
| hsa-miR-23a-5p |
| hsa-miR-24-2-5p |
| hsa-miR-25-3p |
| hsa-miR-25-5p |
| hsa-miR-26a-1-3p |
| hsa-miR-26a-5p |
| hsa-miR-26b |
| hsa-miR-26b-3p |
| hsa-miR-26b-5p |
| hsa-miR-27a-3p |
| hsa-miR-27b-3p |
| hsa-miR-27b-5p |
| hsa-miR-28-3p |
| hsa-miR-28-5p |
| hsa-miR-302c-3p |
| hsa-miR-30a-3p |
| hsa-miR-30a-5p |
| hsa-miR-30b-5p |
| hsa-miR-30c-5p |
| hsa-miR-30d-3p |
| hsa-miR-30d-5p |
| hsa-miR-30e-3p |
| hsa-miR-320a |
| hsa-miR-320b |
| hsa-miR-323a-3p |
| hsa-miR-328-3p |
| hsa-miR-331-3p |
| hsa-miR-335-3p |
| hsa-miR-335-5p |
| hsa-miR-338-5p |
| hsa-miR-33a-5p |
| hsa-miR-340-3p |
| hsa-miR-340-5p |
| hsa-miR-342-3p |
| hsa-miR-345-5p |
| hsa-miR-346 |
| hsa-miR-34a-3p |
| hsa-miR-34b-3p |
| hsa-miR-374b-3p |
| hsa-miR-374b-5p |
| hsa-miR-375 |
| hsa-miR-376c-3p |
| hsa-miR-378 |
| hsa-miR-380-5p |
| hsa-miR-409-3p |
| hsa-miR-411-3p |
| hsa-miR-423-5p |
| hsa-miR-425-3p |
| hsa-miR-425-5p |
| hsa-miR-432-3p |
| hsa-miR-432-5p |
| hsa-miR-450b-3p |
| hsa-miR-451a |
| hsa-miR-454-3p |
| hsa-miR-483-3p |
| hsa-miR-483-5p |
| hsa-miR-484 |
| hsa-miR-485-3p |
| hsa-miR-486-5p |
| hsa-miR-488-5p |
| hsa-miR-495-3p |
| hsa-miR-505-5p |
| hsa-miR-516a-3p |
| hsa-miR-518d-3p |
| hsa-miR-519b-3p |
| hsa-miR-520c-3p |
| hsa-miR-520d-3p |
| hsa-miR-520d-5p |
| hsa-miR-539-5p |
| hsa-miR-543 |
| hsa-miR-548a-3p |
| hsa-miR-550a-3p |
| hsa-miR-550a-5p |
| hsa-miR-564 |
| hsa-miR-571 |
| hsa-miR-572 |
| hsa-miR-573 |
| hsa-miR-574-3p |
| hsa-miR-582-3p |
| hsa-miR-583 |
| hsa-miR-584-5p |
| hsa-miR-593-5p |
| hsa-miR-595 |
| hsa-miR-597-5p |
| hsa-miR-600 |
| hsa-miR-601 |
| hsa-miR-603 |
| hsa-miR-605-5p |
| hsa-miR-623 |
| hsa-miR-624-5p |
| hsa-miR-625-3p |
| hsa-miR-628-3p |
| hsa-miR-628-5p |
| hsa-miR-629-3p |
| hsa-miR-636 |
| hsa-miR-638 |
| hsa-miR-639 |
| hsa-miR-643 |
| hsa-miR-645 |
| hsa-miR-652-3p |
| hsa-miR-659-3p |
| hsa-miR-660-5p |
| hsa-miR-661 |
| hsa-miR-664a-3p |
| hsa-miR-668-3p |
| hsa-miR-675-5p |
| hsa-miR-7-1-3p |
| hsa-miR-708-5p |
| hsa-miR-720 |
| hsa-miR-744-3p |
| hsa-miR-744-5p |
| hsa-miR-758-3p |
| hsa-miR-765 |
| hsa-miR-766-3p |
| hsa-miR-769-5p |
| hsa-miR-770-5p |
| hsa-miR-873-5p |
| hsa-miR-875-5p |
| hsa-miR-885-5p |
| hsa-miR-888-3p |
| hsa-miR-892b |
| hsa-miR-92a-3p |
| hsa-miR-93-3p |
| hsa-miR-93-5p |
| hsa-miR-942-5p |
| hsa-miR-96-3p |
| hsa-miR-99b-3p |
| hsa-miR-99b-5p |

**Supplementary Table 5. MiRNAs detected in plasma pools collected from both groups at baseline (R-t_0_, NR-t_0_) and after 15 months of sitagliptin (R-t_15_ and NR-t_15_)**

| microRNAs |
| --- |
| hsa-let-7b-5p |
| hsa-let-7d-5p |
| hsa-let-7e-5p |
| hsa-mir-103a-3p |
| hsa-mir-106a-5p |
| hsa-mir-10b-3p |
| hsa-mir-1180-3p |
| hsa-mir-1183 |
| hsa-mir-1208 |
| hsa-mir-122-5p |
| hsa-mir-1225-3p |
| hsa-mir-1226-5p |
| hsa-mir-1233-3p |
| hsa-mir-1247-5p |
| hsa-mir-1249 |
| hsa-mir-1252-5p |
| hsa-mir-1253 |
| hsa-mir-126-3p |
| hsa-mir-126-5p |
| hsa-mir-1260a |
| hsa-mir-1274a |
| hsa-mir-1274b |
| hsa-mir-1275 |
| hsa-mir-1276 |
| hsa-mir-1286 |
| hsa-mir-1290 |
| hsa-mir-1291 |
| hsa-mir-1300 |
| hsa-mir-1305 |
| hsa-mir-139-5p |
| hsa-mir-142-3p |
| hsa-mir-144-3p |
| hsa-mir-144-5p |
| hsa-mir-146a-5p |
| hsa-mir-146b-5p |
| hsa-mir-150-5p |
| hsa-mir-151a-3p |
| hsa-mir-151a-5p |
| hsa-mir-155-5p |
| hsa-mir-16-5p |
| hsa-mir-17-5p |
| hsa-mir-181a-2-3p |
| hsa-mir-1825 |
| hsa-mir-183-3p |
| hsa-mir-185-5p |
| hsa-mir-186-5p |
| hsa-mir-190b |
| hsa-mir-191-5p |
| hsa-mir-192-5p |
| hsa-mir-195-5p |
| hsa-mir-197-3p |
| hsa-mir-199b-3p |
| hsa-mir-19a-3p |
| hsa-mir-19b-1-5p |
| hsa-mir-19b-3p |
| hsa-mir-20a-5p |
| hsa-mir-20b-5p |
| hsa-mir-21-3p |
| hsa-mir-21-5p |
| hsa-mir-212-3p |
| hsa-mir-22-5p |
| hsa-mir-222-3p |
| hsa-mir-222-5p |
| hsa-mir-223-3p |
| hsa-mir-223-5p |
| hsa-mir-23a-5p |
| hsa-mir-25-3p |
| hsa-mir-26a-5p |
| hsa-mir-26b-3p |
| hsa-mir-26b-5p |
| hsa-mir-26b |
| hsa-mir-27a-3p |
| hsa-mir-30a-3p |
| hsa-mir-30a-5p |
| hsa-mir-30b-5p |
| hsa-mir-30c-5p |
| hsa-mir-30d-3p |
| hsa-mir-30d-5p |
| hsa-mir-30e-3p |
| hsa-mir-320a |
| hsa-mir-320b |
| hsa-mir-323a-3p |
| hsa-mir-328-3p |
| hsa-mir-335-3p |
| hsa-mir-335-5p |
| hsa-mir-338-5p |
| hsa-mir-342-3p |
| hsa-mir-346 |
| hsa-mir-34a-3p |
| hsa-mir-34b-3p |
| hsa-mir-374b-3p |
| hsa-mir-375 |
| hsa-mir-378 |
| hsa-mir-411-3p |
| hsa-mir-423-5p |
| hsa-mir-425-3p |
| hsa-mir-425-5p |
| hsa-mir-432-3p |
| hsa-mir-451a |
| hsa-mir-483-3p |
| hsa-mir-483-5p |
| hsa-mir-484 |
| hsa-mir-485-3p |
| hsa-mir-486-5p |
| hsa-mir-488-5p |
| hsa-mir-495-3p |
| hsa-mir-505-5p |
| hsa-mir-516a-3p |
| hsa-mir-518d-3p |
| hsa-mir-520c-3p |
| hsa-mir-520d-3p |
| hsa-mir-520d-5p |
| hsa-mir-550a-3p |
| hsa-mir-564 |
| hsa-mir-571 |
| hsa-mir-572 |
| hsa-mir-573 |
| hsa-mir-574-3p |
| hsa-mir-584-5p |
| hsa-mir-593-5p |
| hsa-mir-595 |
| hsa-mir-597-5p |
| hsa-mir-600 |
| hsa-mir-601 |
| hsa-mir-603 |
| hsa-mir-605-5p |
| hsa-mir-623 |
| hsa-mir-624-5p |
| hsa-mir-625-3p |
| hsa-mir-628-3p |
| hsa-mir-636 |
| hsa-mir-638 |
| hsa-mir-639 |
| hsa-mir-659-3p |
| hsa-mir-660-5p |
| hsa-mir-661 |
| hsa-mir-664a-3p |
| hsa-mir-675-5p |
| hsa-mir-7-1-3p |
| hsa-mir-708-5p |
| hsa-mir-720 |
| hsa-mir-766-3p |
| hsa-mir-769-5p |
| hsa-mir-770-5p |
| hsa-mir-875-5p |
| hsa-mir-885-5p |
| hsa-mir-888-3p |
| hsa-mir-92a-3p |
| hsa-mir-93-3p |
| hsa-mir-93-5p |
| hsa-mir-99b-3p |
